# Memory behavior requires knowledge structures, not memory stores

**DOI:** 10.3389/fpsyg.2015.01696

**Published:** 2015-11-03

**Authors:** Guillermo Campitelli

**Affiliations:** School of Psychology and Social Science, Edith Cowan UniversityPerth, WA, Australia

**Keywords:** memory behavior, knowledge structures, expertise, short-term memory, retrieval structures

Since the inception of cognitive psychology dominant theories of memory behavior have used the storage metaphor. In the multi-store models (e.g., Broadbent, 1958; Atkinson and Shiffrin, 1968; Baddeley and Hitch, 1974) the memory system comprises one or more short-term memory (STM) stores and a long-term memory (LTM) store. These stores are places where information is located for varying periods of time (i.e., seconds in the STM stores, and minutes to lifetime in the LTM store) and they have varying capacity limits: large for the LTM store, very limited for the STM store—4 to 7 items, see Miller (1956), Broadbent (1958), and Cowan (2001)^1^. Expertise research has shown that experts are able to remember a large amount of information presented immediately before testing their memory (e.g., more than 80 items in Chase and Ericsson, 1982 and in Gobet and Simon, 1996), suggesting that they are superseding the normal capacity limits of the STM store. However, given that this effect only occurs with domain-specific material expertise theoreticians (e.g., Ericsson and Kintsch, 1995; Gobet and Simon, 1996) explained these results in terms of the use of retrieval structures (see explanation below), but they retained the partition between STM and LTM stores.

In this article I adumbrate an alternative explanation that builds upon three sources: (i) the behaviorist conception of memory as behavior (Delaney and Austin, 1998); (ii) models of memory that exclude the STM store (e.g., Nairne, 1992; Fuster, 1997; Neath, 1998; Cowan, 1999; Oberauer, 2002; Conway et al., 2005; McClelland et al., 2010); (iii) Gobet and Simon's (1996) and Ericsson and Kintsch's (1995) emphasis on the role of expertise in memory, and their pioneer theoretical conceptualization of retrieval structures. In the remaining of the article I briefly discuss these three sources, and then I present the alternative explanation and draw some conclusions.

## Memory as behavior

Delaney and Austin (1998)'s article “Memory as behavior” put forward a behaviorist approach to memory, which opposes the cognitive approaches based on stores. It draws from Chase and Ericsson (1982)'s investigation of strategies used by memory experts, including the use of retrieval structures. In the behaviorist approach memory phenomena are not things that happen to passive organisms; instead, organisms are active in producing those phenomena. People use more or less useful strategies to remember things, and these strategies constitute an essential aspect of memory behavior. This contrasts with the cognitive tradition of memory research in which the use of strategies is avoided because they “contaminate” the “pure” capacity of the STM store. From this approach I value the importance of strategies and the criticism to the attempts to measuring a pure memory capacity. However, unlike this approach, my alternative approach incorporates internal structures.

## Eliminating the STM store

Postulating the existence of a STM store implies that this store has different characteristics to the LTM store. Theoreticians consider that in order to estimate the *pure* limits of the STM store the use of strategies should be avoided, for example, by presenting to-be-remembered items very quickly, or by requesting participants to repeat unrelated syllables (e.g., the–the) in order to avoid participants rehearsing items or using more elaborate strategies such as generating images of the items combined with locations (e.g., the method of loci, see Guida and Lavielle-Guida, 2014 for a discussion).

Aiming at eliminating strategies to measure the limited capacity of the STM store is misleading because previous knowledge is an essential aspect of memory behavior; thus, the phenomenon of interest is severely disrupted. I illustrate this with an example. Consider that exercise scientists would like to measure the *pure* vertical jumping capacity of humans. The high jumping competitions in athletics are ruled out because athletes use strategies that “contaminate” the pure jumping capacity such as running following a semi-circled trajectory, using their arms to aid their jump, etc. The pure jumping capacity might be measured with a vertical jump without running. But, how pure is that? People can still use their arms to aid their jump. Requesting participants to use a straitjacket while they are performing their vertical jump would help, but it would not be enough because people can still bend their knees and lower down their bodies before jumping vertically. Moreover, even if we restrict all the movements of all the joints not involved in vertical jumping people can still contract their muscles without producing movement. Summing up, the pure vertical jumping capacity is an illusion.

Likewise, the intellectual straitjackets used to measure pure STM store capacity do not achieve their goal because using previous knowledge is essential to remember things over the short-term. Moreover, previous knowledge participates at different stages of producing memory behavior. As established by the researchers of the Gestalt school of perception (Wertheimer, 1923; Koffka, 1935), we use our previous knowledge to organize stimuli into familiar ways of grouping, regular shapes, and closed contours. At a “purer” level, in a printed sheet of paper we use our knowledge to perceive lines, dots and curves, not arrangements of ink molecules. Consequently, the whole idea of a limited STM store should be abandoned. Remembering things that were experienced a few moments ago always involves the assistance of previous knowledge, strategies, and abilities. Trying to avoid their involvement not only severely disrupts the phenomenon one aims to investigate, but it is also destined to fail.

Many cognitive psychologists have abandoned the concept of the short-term memory store (e.g., Hintzman, 1986; Nairne, 1992; Fuster, 1997; Neath, 1998; Cowan, 1999; Oberauer, 2002; Conway et al., 2005; Versace et al., 2014) proposing that short-term memory is the part of long-term memory that is currently active, or the part of long-term memory that is currently in the focus of attention, or part of the conscious experience. A different approach that also discards the STM store considers memory phenomena as the emergent property of the functioning of a network (e.g., Botvinick and Plaut, 2006; McClelland et al., 2010; Hasson et al., 2015). These conceptions are closer to the view I am expounding here, albeit insufficient. My criticism is that they rarely take into account the strategies and previous knowledge being used to be able to remember things that occur a few seconds ago (but see, MacDonald and Christiansen, 2002).

## Expertise, strategies, and retrieval structures

Chase and Ericsson (1981) trained two university students to improve their performance in a STM task from a normal score of seven digits to more than 80 digits. In my view this finding should have been taken as a refutation of the models including a fixed limited-capacity STM store. However, the authors preferred to maintain the extant models and introduced the concept of retrieval structures to account for the student's exceptional memory performance. These students were athletes, and they used their knowledge of typical running times in different competitions as retrieval structures. For example, the digit sequence 9-5-8 could be associated to the retrieval structure “Usain Bolt's world record” (which is 9.58 s for the 100 meters race). Ericsson and Kintsch (1995) proposed the long-term working memory (LTWM) theory, which generalizes the use of retrieval structures to account for performance in all domains of expertise, including language. They proposed that experts circumvent the limits of the STM store, not by increasing its capacity (which is fixed), but by using their LTM store as a working memory (through the use of retrieval structures).

A different type of retrieval structure was proposed by Gobet and Simon (1996): the template. The difference between the more typical retrieval structures and the templates is that the former are voluntarily developed with the purpose of improving performance in memory tasks, whereas the later are by-products of acquiring expertise in a field. Moreover, templates consist of a core—a configuration of stimuli that frequently appear in the domain of expertise—and slots in which additional information or less frequent stimuli could be added. Gobet and Simon also proposed that the STM store has a limited capacity and that experts circumvent their limits by storing pointers of the templates in the STM store.

These two proposals represented a step forward in the understanding of memory phenomena. However, mainstream research into short-term memory did not pay too much attention to these proposals, probably because mainstream memory researchers are not interested on the influence of the LTM store on the STM store; rather, they are still interested in avoiding such “contamination,” thus they evade studying material in which people possess some degree of expertise.

## The alternative approach

I propose that we should go a step further and consider retrieval structures as the core of the mechanism underlying remembering things experienced moments ago. Before making this proposal let me tweak the concept of retrieval structure. As useful as it is, the concept of retrieval structure has two problems: First, it was developed under the storage metaphor. These structures are called “retrieval” structures because they retrieve information from the LTM store, which is transferred into the STM store. As I indicated earlier, the STM store does not belong in my alternative explanation. Second, these structures do much more than retrieving information. In Gobet and Simon's view these structures are at the core of thinking processes. Therefore, I will refer to these structures as *knowledge structures*.

Now for my explanation, remembering things over the short term is a phenomenon that requires explanation. The way we remember things is not a passive process in which incoming information remain in a store. Remembering things is an active process that starts before the to-be-remembered information is presented. Based on our current goal we activate the most relevant knowledge structures, and we combine them with incoming information. The success in remembering things that occurred a short-term earlier depends on how relevant the knowledge structures are for the task at hand, the amount of information to be remembered, and the time we have to remember this information. In the case we do not seem to have any relevant knowledge structure (e.g., if we are presented with irregular, novel shapes) we use a very simple knowledge structure: a spatial structure with empty locations which could be filled in with incoming information. This knowledge structure coincides with the slotted structure proposed in traditional models of STM (e.g., Luck and Vogel, 1997), but, unlike those models, it is one of a myriad of knowledge structures we can use to try to make sense and/or memorize incoming information. Moreover, such simple knowledge structure is not free of previous knowledge. There is evidence that in memory tasks people use their experience with reading, and tend to fill the slots from left to right (e.g., van Dijck and Fias, 2011; Guida and Lavielle-Guida, 2014) unless their native language is read from right to left (e.g., Zebian, 2005).

Nonetheless, unless one is a baby it is very rare not to possess relevant knowledge. Thus, in most cases we remember things over the short term by activating domain or task specific knowledge structures. The reason why the typical performance in STM tasks seems to be limited is due to the artificial straitjackets typically used to measure memory performance.

## Implications

My explanation has two main implications that, I hope, inspire future memory research: First, the expertise approach to investigate memory behavior should be taken seriously because we all have certain degree of expertise in performing different tasks (e.g., expertise in face recognition, expertise in speaking one language). Second, this view may shed light on the problem of understanding infants' deficient memory behavior. If building knowledge structures is essential to remember things, the reason why infants have poor memory performance might be that they have very few knowledge structures, not because their STM store is not yet developed or that they have insufficient attentional resources.

### Conflict of interest statement

The author declares that the research was conducted in the absence of any commercial or financial relationships that could be construed as a potential conflict of interest.

## Footnotes

^1^Note that for some authors the limitation is in the attentional resources rather than in the capacity of the STM store (e.g., Pashler and Johnston, 1998; Barrouillet and Camos, 2001). Due to space limitations I will not address this approach. Suffice it to say that my criticism applies to both views.
